# Commonalities and differences in the microbiota-metabolism-immune axis dysregulation patterns between fragile X syndrome and autism spectrum disorder

**DOI:** 10.3389/fped.2026.1735468

**Published:** 2026-04-10

**Authors:** Jianen Zhu, Xiaqing Han, Wenying Zhao, Ying Han

**Affiliations:** Department of Pediatrics, Peking University First Hospital, Beijing, China

**Keywords:** autism spectrum disorder, fragile X syndrome, gut microbiota, serum cytokine, serum metabolome

## Abstract

**Introduction:**

Fragile X syndrome (FXS), a leading monogenic cause of autism spectrum disorder (ASD), provides a crucial model for elucidating ASD pathophysiology. However, comparative studies on the Microbiota-Metabolite-Immune (MMI) axis between these disorders are lacking. This study aims to identify shared and distinct MMI dysregulation patterns to uncover underlying neurobiological mechanisms and potential biomarkers.

**Methods:**

In this cross-sectional study, multi-omics analyses were performed, including 16S rRNA sequencing of gut microbiota, untargeted UPLC-MS-based serum metabolomics, and quantification of 13 serum cytokines. Statistical analyses identified differentially abundant taxa, metabolites, and cytokines between the ASD and FXS groups.

**Results:**

16S rRNA sequencing revealed distinct microbial community structures (beta-diversity) and 11 differentially abundant taxa between groups, though alpha-diversity was comparable. Untargeted metabolomics identified 152 significantly altered serum metabolites, with ASD showing upregulation of metabolites involved in caffeine metabolism and steroid hormone biosynthesis. Cytokine profiling showed significantly elevated IL-17A in FXS vs. ASD among the 13 cytokines analyzed.

**Conclusion:**

This study reveals that FXS and ASD share a common dysregulation framework within the MMI axis, yet exhibit distinct disease-specific patterns, supporting the value of FXS as a monogenic model for ASD. The identified differential metabolites and elevated IL-17A in FXS uncover unique underlying pathophysiological mechanisms, thereby providing potential targets for future biomarker discovery and precise interventions.

## 1 Introduction

Autism Spectrum Disorder (ASD) is a neurodevelopmental disorder that is mainly manifested by difficulties in social communication, narrow interests, and repetitive behaviors, affecting more than 1% of people worldwide (1). The diagnosis of ASD mainly relies on observing behavioral symptoms, and often uses some standardized tools, such as Autism Diagnostic Interview-Revised (ADI-R) and Autism Diagnostic Observation Schedule, Second Edition (ADOS-2), combined with detailed growth records. However, these tools have limitations, particularly in individuals with concurrent sensory impairments or severe behavioral disturbances, which can compromise diagnostic accuracy (2). Given the complex and multifactorial etiology of ASD—encompassing both genetic and environmental contributions—elucidating its neurobiological basis remains critical for improving diagnosis and treatment.

Fragile X Syndrome (FXS) represents the most common monogenic cause of intellectual disability and ASD. It results from an abnormal expansion of CGG trinucleotide repeats (>200) in the 5’ untranslated region of the FMR1 gene on the X chromosome, leading to promoter methylation, transcriptional silencing, and subsequent deficiency of Fragile X Messenger Ribonucleoprotein (FMRP) (3). People with FXS usually have moderate to severe intellectual impairment, comprehensive developmental delay, and significant behavioral problems such as social fear or avoidance, attention deficit hyperactivity disorder, and impulsiveness or aggression tendencies (4). The diagnosis of FXS requires molecular genetic testing to check the number of CGG repeats and the methylation of the FMR1 gene. Southern blot combined with PCR is the gold standard. Despite its accuracy in typical cases, challenges remain in detecting rare mutations or fully characterizing methylation patterns, which may result in missed diagnoses (5).

The relationship between FXS and ASD is particularly noteworthy. FXS is considered to be the most important single gene factor leading to ASD (5). Approximately 60% of FXS men and 14% of FXS women also meet the diagnostic criteria for ASD (6, 7). Moreover, individuals with comorbid FXS and ASD exhibit more severe neurodevelopmental phenotypes (8), suggesting shared pathophysiological mechanisms that extend beyond their distinct genetic etiologies.

Beyond traditional genetic and neuroanatomical perspectives, emerging evidence implicates the gut microbiota and its metabolites in neurodevelopment and behavior through the Microbiota-Gut-Brain Axis (MGBA)—a bidirectional communication network linking the gastrointestinal tract and central nervous system (9). Gut microorganisms and their by-products can influence brain activity and behavior via metabolic and immune pathways. Inflammatory cytokines, for instance, may modulate neurotransmitter systems, neuroendocrine responses, and neural circuits, thereby affecting behavior (10).

To date, most MGBA research in ASD has focused on comparisons with neurotypical development, while direct comparative studies between FXS and ASD remain scarce.Such investigations are essential for deepening our understanding of ASD pathogenesis, identifying FXS-specific biomarkers, and developing targeted interventions. Leveraging FXS as a well-defined monogenic framework for studying ASD-related mechanisms offers a unique opportunity to dissect common and distinct biological pathways.

Accordingly, we hypothesize that FXS and ASD share a core framework of dysregulation along the Microbiota-Metabolite-Immune (MMI) axis, yet exhibit etiology-driven differentiation patterns—reflected in distinct alterations in microbiota composition, serum metabolic profiles, and immune factors. To test this hypothesis, we collected fecal and blood samples from 33 children with ASD and 15 genetically confirmed children with FXS (aged 2–8 years) and performed integrated multi-omics analyses, including 16S rRNA sequencing, untargeted serum metabolomics, and multiplex cytokine profiling. This study aims to identify shared and disorder-specific MMI axis signatures, explore potential biomarkers distinguishing FXS from ASD, and evaluate the utility of FXS as an “etiology-driven model” for investigating ASD neurobiology. By systematically comparing these two related yet distinct neurodevelopmental disorders, we seek to provide new insights into their underlying mechanisms and inform future personalized intervention strategies targeting the MMI axis.

## 2 Materials and methods

### 2.1 Participants and clinical data

This cross-sectional study recruited 48 male children with neurodevelopmental disorders (33 in the ASD group, 15 in the FXS group) aged 2–8 years from multiple regions in China. Enrollment assessments were completed at the Department of Pediatrics, Peking University First Hospital.

For FXS children, the inclusion criteria were as follows: (1)Age 2–8 years; (2)Molecular genetic test results showing full mutation of the FMR1 gene with CGG repeats >200; (3)Male; (4)No gastrointestinal treatments, antibiotics, or probiotics were used within 1 month prior to enrollment; (5)Voluntary participation, with guardians providing informed consent.The exclusion criteria for FXS children were: (1)No molecular genetic testing or results showing FMR1 CGG repeats ≤200; (2)Comorbid other genetic metabolic diseases, psychiatric disorders, organic brain diseases, severe liver/kidney diseases, cardiovascular diseases, autoimmune diseases, etc.; (3)Use of medications (including antibiotics, probiotics) or supplements affecting gastrointestinal function within 1 month prior to enrollment; (4)Guardians refusing participation or unable to complete follow-up.

For ASD children, the inclusion criteria were: (1)Age 2–8 years; (2)Diagnosed by at least two qualified clinicians according to DSM-5 criteria for ASD; (3)Male; (4)No gastrointestinal treatments, antibiotics, or probiotics used within 1 month prior to enrollment; (5)Voluntary participation, with guardians providing informed consent.The exclusion criteria for ASD children were: (1)Autism-like manifestations due to other diseases; (2)Comorbid other genetic metabolic diseases, psychiatric disorders, organic brain diseases, severe liver/kidney diseases, cardiovascular diseases, autoimmune diseases, etc.;(3)Use of medications (including antibiotics, probiotics) or supplements affecting gastrointestinal function within 1 month prior to enrollment; (4)Guardians refusing participation or unable to complete follow-up.

A portion of the FXS children in the 3–8-year age range were also included in our earlier metabolomic study (11), which investigated age-related metabolic changes within FXS. In the present study, these participants are analyzed in a new comparative framework against ASD children, with all FXS-ASD comparisons being previously unreported.

The Chinese version of the Griffiths Development Scales-Chinese (GDS-C) was used to systematically assess six core developmental domains: personal-social, hearing-language, hand-eye coordination, performance, motor, practical reasoning. This study was approved by the Ethics Committee of Peking University First Hospital. All patients or their legal representatives signed written informed consent for diagnostic procedures and biobanking for research purposes.

### 2.2 Collection of blood and fecal samples

Nurses drew venous blood after participants fasted for at least 4 h (without food or drink). Blood samples were centrifuged in two steps at 4°C: first at 1,000 rpm for 10 min, and then at 3,000 rpm for 10 min to separate serum. After centrifugation, the serum was extracted and stored frozen at −80°C. for subsequent testing. Fecal samples were collected within three days of participants joining the study. For three consecutive days, parents collected fecal samples at home each morning. Each daily sample (approximately 50–100 mg) was placed into the same disposable collection tube (containing sodium hydroxide, ethylenediaminetetraacetic acid, sodium chloride, dimethyl sulfoxide, etc.). The tube was capped and shaken after each addition, with care taken to avoid urine or water contamination. It was stored in a cool, dry place during the collection period before final storage at −80°C.

### 2.3 Untargeted metabolomic profiling of serum samples

We used Ultra-Performance Liquid Chromatography-Mass Spectroscopy (UPLC-MS) to examine various metabolites in serum samples. In order to ensure the quality of the data, we took 10 μL of each of 48 serum samples and mixed them together to make a pooled QC sample, and then tested the QC sample every 8 samples to see if the instrument was stable. The salting-out method was used to extract metabolites, and the data was collected using reverse-phase LC-MS in positive and negative ion mode. XCMS software (Nonlinear Dynamics, Durham, NC, USA) was then used to detect characteristic peaks, align retention times, and quantify. In order to remove background interference, we deleted characteristic peaks with intensities below 50, 000 in all samples or absent in more than 85% of the samples. The peak area of pooled QC was then used to correct for batch effects. The relative content was then calculated using the Robust Multichip Average (RMA) method of R's preprocessCore package (v1.47.1). Finally, only characteristic peaks with coefficients of variation (CV) less than 30% in QC samples were retained for continued analysis.

Experiments were performed using an UltiMate 3,000 UPLC system (Thermo Fisher Scientific, USA) plus a Q Exactive Orbitrap mass spectrometer (Thermo Fisher Scientific, Waltham, MA, USA). A BEH C8 column (2.1 × 100 mm, 1.7 μm, Waters, USA) was used in the positive ion mode, and an HSS T3 column (2.1 × 100 mm, 1.8 μm, Waters, USA) was used in the negative ion mode. Mobile phase A is 0.1% formic acid aqueous solution, and B is 0.1% formic acid acetonitrile solution. The gradient elution program is: hold 95% A for 0.0–1.0 min, drop to 5% A for 11.0 min, hold 95% B for 11.1–13.0 min, and return to 95% A for 15.0 min. The flow rate was 0.35 mL/min, and the injection volume was 5 μL.

Mass spectrometer settings: Electrospray ionization (ESI) was operated in positive ion mode (+3.8 kV) and negative ion mode (−3.0 kV), the capillary temperature was set to 320°C, the sheath gas flow rate was 35 arbitrary units, the auxiliary gas temperature was 350°C, and the S-lens RF level was maintained at 50. The m/z detection range is 100–1,200 in ESI positive ion mode and 70–1,060 in negative ion mode. The full scan resolution is 70, 000 (FWHM at m/z 200), while the MS/MS scan resolution is 17, 500 (FWHM at m/z 200). The fragmentation uses step collision energies of 20 and 40 normalized collision energies (NCE). Mass calibrations are performed daily using Pierce™ LTQ Velos™ Calibration Standards (Thermo Fisher Scientific) to ensure mass accuracy within 2 ppm.

For quality control samples, MS/MS spectra generate secondary mass spectra by selecting the first 10 precursor ions and applying collision energies of 25 and 50 NCE. These primary and secondary spectra were processed using MS-DIAL version 4.24 and identified by matching with public databases such as HMDB and PubChem. Preliminary annotation of metabolites was achieved by comparing observed retention times, if any, and MS/MS fragmentation patterns with data in the METLIN and HMDB databases. The integration of retention time data and MS/MS spectra produces unique fragment ion signatures that reflect molecular structure, functional groups, and applied collision energies. Structural assignment is accomplished using similarity-based spectral analysis tools such as GNPS, and metabolites are classified based on their functional group composition. Finally, the metabolites were grouped according to their chemical annotation categories.

We used R software (version 4.2.1) to perform data pretreatment, statistical analysis, and predictive models to evaluate the relative concentrations of each metabolite. First, the raw metabolite content data of all samples were normalized using the Robust Multichip Average method. After standardization, we calculated the relative content and used it for subsequent analysis.

Through Analysis of Variation (ANOVA), we identified metabolites with significant differences, with adjusted *p*-values less than 0.05. In addition, we also screened for metabolites with fold changes between groups outside the range of 0.7–1.5. In addition to this fold of change screening (>1.5 or <0.7), we also used the Benjamini-Hochberg False Discovery Rate (FDR) method to perform multiple test corrections, and q values less than 0.05 were accepted to reduce false positive results.

We used MetaboAnalyst 5.0 to conduct a Kyoto Encyclopedia of Genes and Genomes (KEGG) pathway enrichment analysis to map annotated metabolites to the KEGG pathway. The degree of enrichment of pathways was assessed using a hypergeometric test, and pathways with FDR less than 0.05 were considered statistically significant and corrected for multiple tests.

### 2.4 Metagenomic analysis of fecal samples

We used the OMEGA Mag-Bind Soil DNA Kit (M5635–02, Omega Bio-Tek, USA) to extract genomic DNA from each sample. The purified DNA was used to build a macro genomic shotgun sequencing library, with a target insert size of about 400 base pairs. Processing steps of raw sequencing data include: (1 ) using cutadapt to remove primer sequences; (2) using demux to split samples; and (3) using DADA2 plug-in for quality control, noise reduction, sequence merging, and mosaic removal. All libraries were sequenced on the Illumina NovaSeq system (USA) using double-ended 150 bp reads.

After strict quality control, the resulting high-quality sequences were species annotated using the Genome Taxonomy Database (GTDB). Reads from each sample were assembled using mseqs2 (shortest contig 300 bp) and grouped by sequence similarity (95% agreement, shortest coverage 90%). Non-redundant contigs were compared with the NCBI-nt database using mmseqs2, species classifications were assigned and gene abundances were calculated using the lowest common ancestor method.

Differences in species and functional characteristics between groups were compared using the Linear Discriminant Analysis Effect Size (LefSe) method. Differences between groups in microbial community structure and functional capabilities were analyzed for beta diversity using the Bray-Curtis distance matrix.

### 2.5 Multiplex cytokine assay

We used a commercially available test kit (RayBiotech, Guangzhou;RayPlex™ Human Inflammation Array 1, article no. FAH-INF-1-96) that is based on flow cytometry to measure cytokine concentrations in serum. It can measure 13 cytokines at one time: IFN-gamma, IL-4, IL-6, IL-10, IL-17A, TNF alpha, IL-1 beta (IL-1F2), IL-2, IL-12p70, G-CSF, IL-13, IL-23p19 and MCP-1 (CCL2). The entire process is operated in full accordance with the instructions. Serum samples are taken out of freezing and placed on ice to be thawed and used immediately. Simply put, first add magnetic beads to the wells of a 96-well plate, wash the beads, then add RayPlex multiple magnetic bead mixture, and then add serum or standards. Then place the plate on a shaker and shake at 1,000 rpm for 2 h at room temperature. After washing, add the biotin-labeled detection antibody mixture and shake for 1 h at room temperature. Wash twice more, add streptavidin-fluorescein solution to each well, and shake for 30 min at room temperature. Wash it one last time, add reading buffer, and shake for a while more. Fluorescence signals were measured using the Luminex MAGPIX system (software versions xPonent 4.2 and ProcartaPlex Analyst 1.0), and the amount of cytokines was calculated based on a standard curve from a five-parameter logistic regression model. If a certain cytokine is not detected in the sample, the lowest standard concentration is used for calculation; if the highest standard concentration is exceeded, the maximum value is used for calculation.

We calculated concentration values from the original multiplex cytokine data and used these concentration values to continue the analysis. The statistical method uses moderated t-statistics in the limma package (a tool in R/Bioconductor), which uses adjusted *p*-values (BH-corrected) and log fold changes (log2 scale) to find differentially expressed proteins. The screening criteria are: logFC > log2 (1.2), fold change greater than 1.2 fold, and adjusted or raw *p*-value less than 0.05. We plot a scatter plot using ggplot2 and ggfortify packages, with AveExp on the x and y axes (the average of the logarithm in each group). The red marks on the graph are up-regulated proteins, the blue marks are down-regulated, and the gray marks no significant changes.

After finding the differentially expressed proteins, we drew a volcano plot to show the results. This graph shows genes with significant differences in expression between the two groups. The *x*-axis is logFC and the *y*-axis is the negative logarithm of the adjusted or unadjusted *p*-value [-log10 (adj. P. Val or *p*-value)]. The drawing is also done with ggplot2 and ggfortify packages.

## 3 Results

### 3.1 Comparison of GDS-C scores between ASD and FXS children

This study included 33 ASD patients and 15 FXS patients, all of whom completed serum and stool sample collection and GDS-C scale assessment. The GDS-C scale was used to systematically assess core developmental domains: personal-social, hearing-language, hand-eye coordination, performance, motor, practical reasoning. The GDS-C assessment was conducted by a fixed qualified professional strictly following standards in a dedicated assessment room. The assessment yielded the developmental age equivalent for each domain, and the developmental quotient (DQ) was calculated as: DQ = developmental age equivalent/chronological age ×100.A DQ of 100 represents performance at the chronological age level, with scores typically between 85 and 115 considered within the normal range for typically developing children. Normal development: ≥85, borderline: 70–85, developmental delay: <70. Comparison of GDS-C scores between ASD and FXS children indicated (as shown in Table 1) that among the six assessed domains of the GMSD-C, the difference in the hand-eye coordination domain score between the two groups was statistically significant, while scores in the other domains showed no significant differences between the two groups.

**Table 1:** Comparison of GDS-C DQs between children with ASD and children with FXS.

GDS-C Domain	FXS (*n* = 15)	ASD (*n* = 33)	*p*-value	Test statistic (T/Z)
Locomotor, mean(SD), score	56.0 (22.1)	66.6 (17.4)	0.079	−1.798
Personal-social, mean(SD), score	50.7 (23.9)	55.6 (17.2)	0.421	−0.811
Language, mean(SD), score	39.6 (26.0)	50.6 (28.8)	0.214	−1.261
Eye-hand Coordination, mean(SD), score	44.8 (21.4)	58.3 (18.2)	0.029	−2.250
Performance, mean(SD), score	50.7 (27.2)	63.7 (18.8)	0.060	−1.926
Practical reasoning, median (IQR), score	31.7 (0–61.0)	35.0 (0–75.2)	0.551	−0.597

The table presents score statistics for the two groups of children in the GDS-C personal-social, hearing-language, hand-eye coordination, performance, motor, and practical reasoning dimensions. Data satisfying normal distribution are presented as mean and standard deviation (SD); data not satisfying normal distribution are presented as median and interquartile range. Independent samples t-test and non-parametric tests show the test statistic (T value or Z value) and significance level (*P* value) for intergroup differences. *p* < 0.05 indicates a statistically significant difference.

FXS, fragile X syndrome; ASD, autism spectrum disorder; GDS-C, The Griffiths Development Scales-Chinese.

### 3.2 Comparison of gut microbiota characteristics between ASD and FXS children

**Figure 1:**
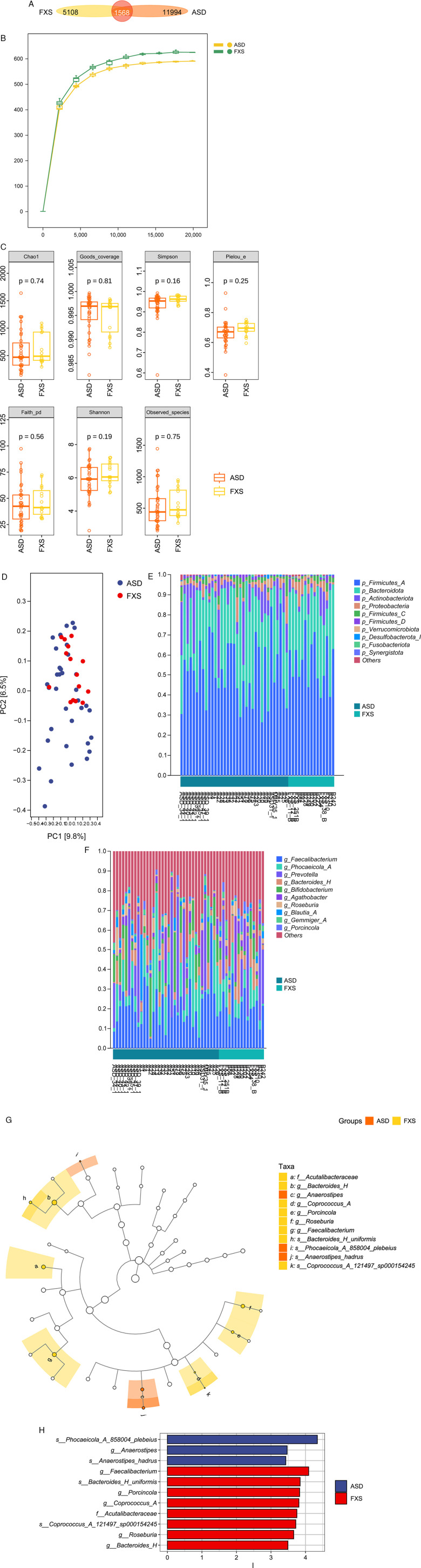
Gut microbiota analysis results for the ASD group (*n* = 33) and FXS group (*n* = 15). **(A)** This Venn diagram shows the number of ASVs in different sample groups, marking their unique and common parts. **(B)** The dilution curve for each sample was drawn by randomly selecting sequences to show the number of species we saw: different sequencing depths greatly affect the diversity detected. When the curve flatters, sequencing is sufficient and represents the species richness of this sample. The flatter the curve, the more sequencing reflects the true diversity of the samples. **(C)** Alpha diversity indicators include Chao1, Observed species, Shannon and Simpson indices, which are used to describe the richness and diversity of microbiota. **(D)** Principal coordinate analysis (PCoA) based on the Bray-Curtis distance showed differences in the structure of microbial communities between the two groups. **(E)** Stacked bar graphs show the composition of the gut flora at the phylum level. **(F)** Stacked bar graphs show the composition of the gut flora at the genus level. **(G)** The cladogram obtained through LefSe analysis identifies taxa with significant differences between the two groups. **(H)** The bar chart shows the LDA scores of the species with significant differences. ASD, autism spectrum disorder; FXS, fragile X syndrome; ASV, amplicon sequence variants; LefSe, linear discriminant analysis effect size; LDA, linear discriminant analysis.

This study aimed to investigate the shared characteristics and key differences in the gut microbiota of children with ASD and those with FXS. We collected fecal samples from 33 children with ASD and 15 children with FXS, which were analyzed using 16S rRNA amplicon sequencing technology. During the preprocessing of sequencing data, the demux plugin was first used for sample demultiplexing, followed by primer sequence removal using the cutadapt plugin. Subsequently, bioinformatics tools including DADA2 were applied for quality control, denoising, sequence merging, and chimera removal. Processed sequences were clustered into feature Amplicon Sequence Variants (ASVs) and an ASV abundance table was constructed under a 100% sequence similarity threshold. The results identified a total of 18, 670 ASVs, with 1, 568 ASVs overlapping between the ASD and FXS groups (Figure 1A). The number of ASVs unique to the ASD group was substantially greater than that unique to the FXS group, indicating a richer gut microbiota in the ASD group. Furthermore, rarefaction curves were plotted based on species-based ASV and sequence counts per sample. The slope of these curves effectively reflects the impact of sequencing depth on diversity estimates and serves as an important indicator of species richness within samples. The plateauing of the rarefaction curves suggests that the sequencing depth adequately captured the microbial diversity, indicating sufficient sampling (Figure 1B).

Alpha diversity measured using Chao1, Observed species, Shannon, Simpson, and Faith's PD indicators showed no significant differences in species richness or diversity between the two groups (Figure 1C). Comparing the Beta diversity of microbial community structure between samples using Bray-Curtis distance found that ASD and FXS partially overlapped on the PC1-PC2 axis but had significantly different clustering patterns (*p* = 0.02) (Figure 1D). We also studied the relative abundance of intestinal flora at the phylum and genus levels, focusing on the 10 most common groups (Figures 1E,F). At the portal level, p_Firmicutes_A, p_Bacteroidota, p_Actinobacteria and p_Proteobacteria were dominant in both ASD and FXS groups. At the genus level, the main groups include g_*Faecalibacterium*, g_*Phocaeicola_A*, g_*Prevotella* and g_*Bacteroides_H*. Analysis using LefSe found that there were significant differences between the ASD and FXS groups (*p* < 0.05, LDA > 3.4). Specifically, the relative abundances of s_*Phocaeicola_A_858004_plebeius*, g_*Anaerostipes*, s_*Anaerostipes_hadrus*, etc. were significantly higher in the ASD group, while the levels of g_*Faecalibacterium*, s_*Bacteroides_H_uniform*, g_*Porcinicola*, etc. were significantly higher in the FXS group (Figures 1G,H).

### 3.3 Comparison of serum metabolite characteristics between ASD and FXS children

**Figure 2:**
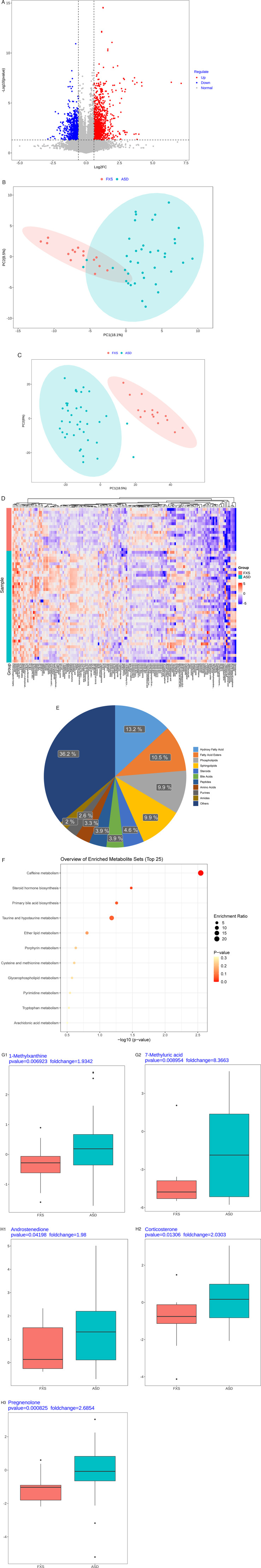
Serum untargeted metabolomics analysis results for the ASD group (*n* = 33) and FXS group (*n* = 15). **(A)** Abundance of metabolites significantly altered between groups in RPLC-MS analysis (FXS vs. ASD, *p* < 0.05, fold change >1.5 or <0.7). **(B)** PCA plot based on all altered metabolites. **(C)** PCA plot based on annotatable altered metabolites. **(D)** Heatmap of marked metabolites among the differential metabolites. **(E)** Pie chart showing the category composition based on the number of altered metabolites. **(F)** Bubble chart of KEGG pathway enrichment analysis for metabolites with specific compound annotations. **(G1-G2)** Differential expression of 1-methylxanthine and 7-methyluric acid between FXS and ASD groups. **(H1-H3)** Differential expression analysis of androstenedione, corticosterone, and pregnenolone between FXS and ASD groups. RPLC-MS, reversed-phase liquid chromatography-mass spectrometry; PCA, principal componentanalysis; KEGG, Kyoto Encyclopedia of Genes and Genomes.

Serum samples from 48 individuals were collected and subjected to untargeted metabolomic analysis. After strict quality control (raw abundance >50, 000 in all QC samples and CV < 30%), 43, 021 reliable feature peaks were detected. After FDR correction using the Benjamini-Hochberg method (significance threshold FDR < 0.05), combined with a biological change threshold [|log2(Fold Change)| > log2(1.5)], a total of 2, 307 significantly differentially expressed feature peaks were screened (Figure 2A). Among these, 152 feature peaks were successfully annotated to specific metabolites (Figures 2B,C). Compared to the FXS group, 115 of these annotated metabolite features were increased in abundance and 37 were decreased in the ASD group. The relative abundances of these annotated metabolites are shown in Figure 2D. Analysis of the categories of the 115 metabolites showed that, compared to the FXS group, metabolite concentration changes in the ASD group were mainly concentrated in hydroxy fatty acids, fatty acid esters, phospholipids, and sphingolipids (Figure 2E). Specifically, fatty acid esters, phospholipids, and sphingolipid metabolites were elevated in the ASD group serum, while hydroxy fatty acid metabolites were higher in the FXS group (Supplementary Table S3).

To further elucidate the biological significance of the differential serum metabolome between the ASD and FXS groups, differentially expressed metabolites with clear compound annotations were selected for KEGG pathway enrichment analysis. The results showed that, compared to the FXS group, upregulated metabolites in the ASD group were significantly enriched in two pathways: caffeine metabolism and steroid hormone biosynthesis (Figure 2F). Within these pathways, 1-methylxanthine and 7-methyluric acid in the caffeine metabolism pathway (Figures 2G1-G2), and androstenedione, corticosterone, and pregnenolone in the steroid hormone biosynthesis pathway (Figures 2H1-H3), all showed an upward trend in the ASD group relative to the FXS group. These results reveal group-specific differences in serum metabolic features between ASD and FXS.

### 3.4 Comparison of serum cytokine characteristics between ASD and FXS children

**Figure 3:**
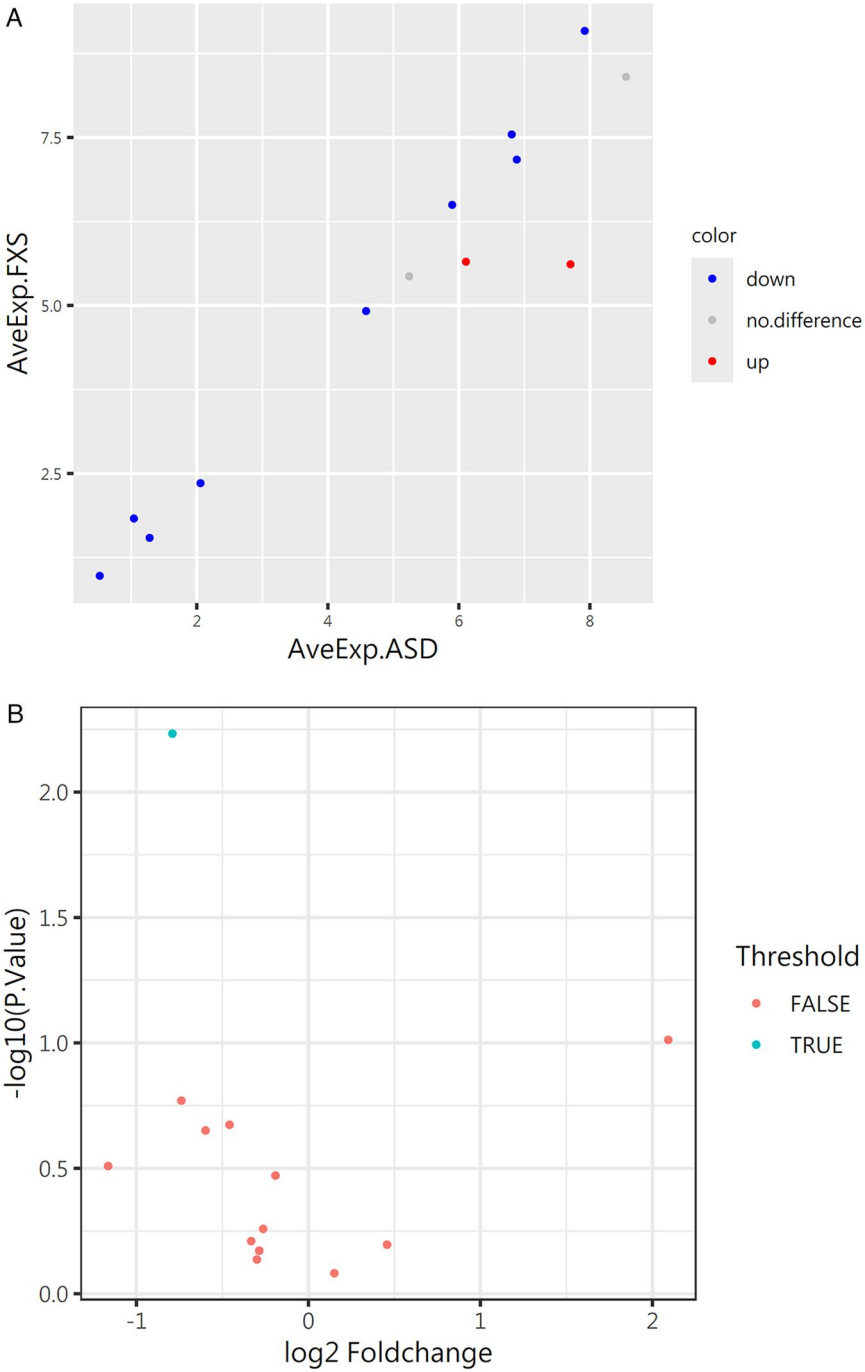
Serum levels of 13 cytokines in the ASD group (*n* = 33) and FXS group (*n* = 15). **(A)** The horizontal and vertical axes represent the average cytokine expression levels in the ASD and FXS groups, respectively. Red indicates upregulated proteins, blue indicates downregulated proteins, gray indicates non-significant differences. **(B)** Volcano plot visualization after screening for differential proteins.

This study measured the levels of serum cytokines in ASD and FXS children, including IFN-gamma, IL-4, IL-6, IL-10, IL-17A, TNF alpha, IL-1 beta (IL-1F2), IL-2, IL-12 p70, G-CSF, IL-13, IL-23 p19, MCP-1 (CCL2). The study found that although the levels of most cytokines in ASD patient serum were downregulated compared to FXS (Figure 3A), the cytokine with a statistically significant difference was IL-17A (Figure 3B).

## 4 Discussion

This study is the first to systematically compare the characteristics of the MMI Axis in children with FXS and ASD by integrating data on gut microbiota, serum metabolome, and immune factors. We acknowledge that the role of the MMI axis in FXS and its direct comparison with ASD represents an emerging area of investigation with currently limited evidence. Our study is exploratory in nature, aimed at generating hypotheses and providing preliminary data to inform future confirmatory research. Accordingly, the findings presented here should be interpreted with appropriate caution and require validation in larger, independent cohorts.

Although limited by sample size and the lack of a normal control group, we found that against a background of largely similar developmental-behavioral phenotypes, FXS and ASD exhibit a pattern of “disease-specific differentiation within a common framework” on the MMI axis. Previous studies analyzing resting-state functional magnetic resonance imaging (fMRI) data and GDS-C data from children with ASD and FXS have confirmed that these two disorders share common features but also have unique differences in brain network architecture (12). Interestingly, our study further reveals a similar “commonality-specificity” pattern along the MMI axis—both groups share a broadly similar framework while also exhibiting disease-specific characteristics. These findings provide new perspectives for understanding the heterogeneous mechanisms of the two disorders. It is important to note that the above conclusions focus on the relative differences between the two disorders, and all findings should be considered preliminary and exploratory.

Comparison of GDS-C scores between children with ASD and those with FXS revealed a statistically significant difference only in the hand-eye coordination dimension (FXS group scores were significantly lower than the ASD group), with no significant differences in other assessed domains of the scale (Table 1). This suggests that although the molecular pathogenesis of FXS and ASD differs, both disorders may ultimately lead to similar developmental phenotypes by regulating shared neurodevelopmental pathways. The difference in hand-eye coordination scores might reflect unique details in neural pathway regulation specific to each disorder, providing clues for exploring disease-specific intervention targets.

Despite fundamental etiological differences between FXS and ASD, they already show partially similar dysregulation patterns at the MMI axis level. Focusing further on the key regulatory level of gut microbiota, this study found that the microbial characteristics of the two disorders also exhibit “coexisting commonality and specificity": Firstly, both the ASD and FXS groups shared common functional disturbances in gut microbial communities, suggesting they may share a similar basis of microbial dysbiosis. Secondly, in the 16S rRNA amplicon sequencing results, the number of unique ASVs was slightly higher in the ASD group than in the FXS group (Figure 1A), suggesting higher diversity of gut microbiota in children with ASD—a conclusion supported by rarefaction curve results. In terms of overall community structure differences, beta-diversity analysis showed a “partially overlapping yet distinguishable” clustering trend between ASD and FXS samples (Figure 1D): the overlapping areas further indicate potential associations between the two groups’ microbiota, while the dispersed distribution reflects heterogeneity within each group. Furthermore, detailed analysis at different taxonomic levels showed no significant difference in microbial composition abundance at the phylum level between the FXS and ASD groups (Figure 1E), but clear differences in composition proportions were observed at the more refined genus level (Figure 1F).

To contextualize our clinical findings and assess their translational relevance, we compared the gut microbiome signatures observed in our ASD and FXS cohorts with data from established preclinical animal models. Our clinical data indicate that Firmicutes, Bacteroidetes, Actinobacteria, and Proteobacteria were the dominant phyla, a finding consistent with the overall compositional framework reported in classic ASD models—such as the BTBR T + tf/J (BTBR) mouse and the valproic acid (VPA)-exposed rodent model—as well as in the Fmr1 knockout (KO) mouse model of FXS (13–15). However, a more granular comparison at the genus or species level reveals both convergences and divergences, highlighting the complexity of translational recapitulation. For instance, the decrease in the abundance of short-chain fatty acid (SCFA)-producing bacteria such as Faecalibacterium reported in some ASD models (e.g., VPA-exposed rats) partially aligns with our observation of a lower relative abundance of Faecalibacterium in the ASD group compared to the FXS group (14). This suggests that models capturing a deficit in beneficial SCFA producers may possess construct validity for certain aspects of ASD pathophysiology. Conversely, some model-specific findings were not reflected in our human cohort: prominent shifts in Akkermansia or Bifidobacterium reported in Fmr1 KO mice were not core differentiating features in our human FXS cohort, where Sutterella was significantly elevated compared to the ASD group (15). Notably, several bacterial taxa identified as significantly discriminant in our clinical study—such as Tannerella forsythia A group and Anaerorhabdus in the ASD group, and Bacteroides vulgatus H group in the FXS group—have not been consistently highlighted in the current preclinical literature. This discrepancy may stem from species-specific host-microbe interactions, differences in environmental exposures (e.g., diet, antibiotic use), or the inherent limitations of using a single genetic or induced animal construct to model a heterogeneous human disorder.

The serum metabolic profiles revealed disease-specific patterns between children with FXS and ASD. Compared to the FXS group, the ASD group exhibited upregulation in caffeine metabolism and steroid hormone biosynthesis pathways, along with elevated levels of fatty acid esters, phospholipids, and sphingolipids, suggesting more active lipid metabolic reprogramming and distinct neuroendocrine regulation. A key finding was the significant upregulation of pregnenolone in ASD relative to FXS. Given the absence of a healthy control group, this relative difference could arise from an elevation in ASD, a reduction in FXS, or a combination. Notably, FXS is consistently linked to a deficiency in allopregnanolone, a downstream neuroactive metabolite of pregnenolone (16, 17). Thus, a parsimonious interpretation is that the observed difference may primarily reflect an inhibition of pregnenolone synthesis or conversion in FXS. This provides an upstream mechanistic clue for the known neurosteroid deficiency in FXS. Given that GABAergic system dysfunction is a core pathophysiological feature of FXS, targeting this pathway with allopregnanolone, its synthetic analogs, or upstream precursors like pregnenolone holds significant therapeutic potential. Preclinical studies have confirmed that these neurosteroids can restore excitatory/inhibitory (E/I) balance, reverse neuronal hyperexcitability, and improve behavioral phenotypes in FXS models (18). Supporting this rationale, a preliminary open-label trial investigating allopregnanolone for Fragile X-associated Tremor/Ataxia Syndrome (FXTAS) reported potential improvements in some cognitive deficits, subtle positive effects on MRI metrics in a subset of patients, and a favorable tolerability profile (19). This mechanistic link supports the rationale for related replacement therapies (e.g., allopregnanolone analogs such as ganaxolone).

Analysis of 13 serum cytokines indicated that except for a significantly higher level of serum IL-17A in the FXS group compared to the ASD group, the other 12 cytokines showed no statistically significant differences between the two groups (Figure 3B). This result suggests that FXS and ASD share similar regulatory patterns for most immune indicators, while the specific elevation of IL-17A might serve as a potential biomarker distinguishing FXS from ASD.

The small sample size of the FXS group in this study may be insufficient to capture within-group heterogeneity (e.g., the impact of comorbidities like epilepsy or anxiety on the MMI axis), potentially leading to an underestimation of some subtle common signals; it also limited the ability to explore subgroup analyses (e.g., FXS with/without ASD). Secondly, the lack of a neurotypical (NT) development control group means this study cannot determine whether the MMI axis features are specific to the disease state or are general phenomena of the developmental stage, making it difficult to quantify the degree of “dysregulation.” We plan to expand the sample size and include an NT group in the future to establish healthy reference ranges for MMI axis parameters and further validate the results obtained here. Additionally, stratification based on comorbidities (epilepsy, anxiety) and ASD co-diagnosis status could improve the precision of subgroup analysis.

## 5 Limitations of the study

A major limitation of the present study is the lack of an internal neurotypical control group. Furthermore, systematic literature searches failed to identify age-matched, methodologically consistent external normative reference data that align with our sample type and multi-omics detection platforms, which precluded cross-study comparisons with healthy children aged 2–8 years. Consequently, the present analysis was restricted to inter-group differences between FXS and ASD cohorts, and we were unable to directly characterize the deviations of their relevant indices from the healthy developmental baseline.

In addition to the absence of a healthy control group, several methodological and confounding factors should be considered when interpreting our findings. Firstly, this study did not collect detailed dietary records, information on food allergies or avoidances, or comprehensive medication histories, which are important factors that can influence the gut microbiome and metabolome. The absence of these data limits our ability to control for these potential confounders.

A key methodological consideration among these is the washout period for antibiotics and probiotics. Although a standardized one-month period was applied, contemporary evidence suggests this duration is likely insufficient for complete restoration of the gut microbiome (20, 21). Consequently, our findings may partially reflect long-term alterations of the gut ecosystem from prior exposures, rather than representing a fully restored baseline state. This necessitates cautious interpretation of the observed microbial differences between groups. Therefore, the results presented here should be interpreted as characterizing the current-state microbiome under the defined washout conditions, not its post-recovery state.

Relatedly, our study did not specifically account for the higher predisposition to recurrent infections, such as otitis media, in children with FXS. This likely results in greater cumulative lifetime antibiotic exposure in the FXS cohort compared to the ASD cohort. Although we excluded recent antibiotic use, we did not collect detailed historical data on the frequency, duration, or types of past antibiotic courses or infections. Consequently, we could not quantify or control for this potential source of long-term microbial perturbation. The lack of this historical data limits our ability to determine whether observed differences in the gut microbiome are directly linked to the core neurodevelopmental pathology of FXS or are partially mediated by its associated infectious comorbidities and their treatments.

Beyond antibiotics, a notable limitation stems from the detection of various exogenous pharmaceutical compounds in our untargeted metabolomic data. These included medications such as gabapentin, famotidine, oxibendazole, albendazole, and ethionamide, which are likely prescribed for common comorbidities (e.g., irritability, gastroesophageal reflux, or infections) in this clinical pediatric cohort. While their presence reflects the real-world clinical complexity of the studied populations, these compounds constitute potential, uncontrolled confounders. They may directly or indirectly modulate gut microbiome composition and host metabolism, thereby influencing the observed inter-group differences between ASD and FXS. Although our study controlled for recent use of antibiotics and probiotics, we did not exclude participants based on stable, long-term pharmacotherapies to maintain clinical representativeness and feasibility. Consequently, the specific contributions of disease pathophysiology vs. medication effects to the MMI axis profiles cannot be fully disentangled in the present study.

Finally, our study has methodological considerations regarding fecal sampling. Samples were collected in the early morning to minimize diurnal variation, although the exact time was not recorded, which may add noise to the data. Additionally, we used a single-aliquot ’spot sample’ without homogenizing the entire stool specimen. While pragmatic, this approach may not fully capture spatial microbial heterogeneity and could increase variability, especially for low-abundance taxa.These factors, combined with inherent interindividual differences in microbiome composition, underscore the need for cautious interpretation and validation in larger cohorts. Future studies with precisely timed collection and homogenized samples would help reduce these sources of variation.

Together, these limitations highlight directions for future research. This limitation will be addressed in future research by enrolling a large sample of age- and gender-matched typically developing children as internal controls and conducting multi-center collaborative studies. Such efforts will validate the inter-group differences identified in this study, clarify the specific deviations of relevant indices in FXS and ASD from the healthy baseline, and provide more robust evidence for elucidating the pathogenic mechanisms of these disorders and developing targeted intervention strategies. Furthermore, future rigorously controlled studies should incorporate detailed, prospective medication logging to allow for covariate adjustment or stratified analyses, or ideally include medication-naïve participants, to better isolate the core disease-specific signatures.

## Data Availability

The original contributions presented in the study are publicly available. This data can be found here: MetaboLights (accession: MTBLS14135) and NCBI Sequence Read Archive (accession: PRJNA1208259).
